# Supplementary material for: Intra‐arterial transplantation of HLA‐matched donor mesoangioblasts in Duchenne muscular dystrophy
Source: EMBO Mol Med. 2015 Nov 5;7(12):1513–28. doi: 10.15252/emmm.201505636 (PMC4693504; doi:10.15252/emmm.201505636)

Table of Contents:

Appendix Tables S1 to S4
Appendix Figures S1 to S9

Appendix Table S1: Phenotype of donor cells

	Donor 01	Donor 02	Donor 03	Donor 05	Donor 06
Senescence:<br>% of total cells	2.7*	2.8	3.7	4.3	1.0
Myogenesis:<br>% of total cells	25*	18	38	23	24
Viability at infusion	96-99°	92-97	94-99	97-98	93-99
Cell cycle:<br>% of cells in S phase	3-13	2-16	3-14	2-4	4-8
CD31 % of total cells	0.1-0.3	0	0.1-0.5	0	0
CD34 % of total cells	0	0-0.3	0-0.2	0	0-1
CD45 % of total cells	0	0	0-0.1	0	0
CD13 % of total cells	98-99	99-100	98-99	100	100
CD44 % of total cells	90-99	99-100	98-99	100	98-100
CD56 % of total cells	2-6	3-5	1-4	2-3	4-6
ALP % of total cells	2-4	3-17	2-11	8-11	15-27

Legend: The table shows the features of the donor cell populations used in the study. The Donor number matches the number of the sibling patient. * Refers to parameters measured on the IP before freezing. ° Refers to parameters measured on the MP before infusions. Range (min-max) for the four infusions are reported.

Appendix **Table S2: Adverse events**

**Pt01**	**Livedo Reticularis**	**Infections**	**Electrolyte dysbalance**	**Hypertension**	**Other AE**
Month 0 to 12 (March 2011-May 2012)	After 1st MAB Infusion (March 2011)	- Cough (November 2011)<br><br>- Pitiriasi Versicolor (March-May 2012)	Mild hypo-magnesemia (March-April 2011)	January 2012	- Semisolid stools (candida positive) (March-April 2011)<br><br>- Femoral fracture after accidental fall (May 2012)
Therapy (FK506 stopped in June 2013)	None	- Oral Antibiotic (November 2011)<br><br>- Topic antifungal treatment (March-May 2012)	Oral administration (March-April 2011)	ACE-Inhibitors (from March 2012)	Orthopedic brace and Heparin
**Pt02**	**Livedo**	**Infections**	**Electrolyte**	**Hypertension**	**Other AE**

	Reticularis		unbalance		
Month 0 to 12 (April 2011- June 2012)	After 3rd and 4th MAB Infusion (October 2011 and December 2011)	- type A Flu (February 2012)<br><br>-Streptococcus pharyngitis (July - August 2012)	Hypo-magnesemia (December 2011)	None	- Semisolid stools and abdominal pain (June 2012)
Therapy (FK506 stopped in July 2014)	None	- Oral antiviral and antibiotic	Oral supplementation	None	None
After month 12 (Additional infusion December 2012)	None	Viral gastroenteritis (February 2013)	None	None	Loss of autonomous ambulation (January 2013)
**Pt03**	**Livedo Reticularis**	**Infections**	**Electrolyte dysbalance**	**Hypertension**	**Other AE**
Month 0 to 12 (May 2011 - July 2012)	None	Pityriasis versicolor (December 2011)	Hypo-magnesemia (from May 2011)	None	- Arterial Vasospasm after 1st catheterization (May 2011)<br><br>- Low back pain (December 2011)<br><br>- Atrial Fibrillation and thalamic stroke after 4th MAB infusion (December 2011)<br><br>- Loss of autonomous ambulation (June 2012)
Therapy (FK506 stopped in June 2013)		Topic antifungal treatment (December 2011)	Oral administration (May 2011)		-Aspirin (from December 2011)
After month 12	None	None	None	ACE-Inhibitors	- Metabolic Syndrome and Obesity<br><br>- Steroid stopped in October 2013
**Pt05**	**Livedo Reticularis**	**Infections**	**Electrolyte unbalance**	**Hypertension**	**Other AE**
Month 0 to 12 (November 2012 - December 2013)	None	Fever and cough (January 2013)	Hypo-magnesemia (May 2013)	None	- Accidental fall (October 2013)

Therapy		Oral antibiotic	Oral administration		
After Month 12 (additional infusion February 2014 and April 2014)	None	None	None	None	- Anemia (February 2014) oral iron therapy<br><br>- Stools positive for Helicobacter Pylori (eradication therapy)
**Pt06**	**Livedo Reticularis**	**Infections**	**Electrolyte dysbalance**	**Hypertension**	**Other AE**
Month 0 to 12 (December 2012 - December 2013)	None	- Fever December 2012	None	None	None
Therapy		Oral antibiotic			

Legend. The rate of infections in the treated patients appeared comparable to healthy patients of pediatric age relatively to the viral and bacterial infections, but slightly increased relatively to fungal infections. All the pattern of the reported infections is consistent with the use of a double immunosuppressive treatment (tacrolimus and chronic steroids).
The infections were mostly viral or bacterial, occurring in winter time (cough in Pt 01; type A Flu, viral gastroenteritis and Streptococcus pharyngitis in Pt 02; fever and cough in Pt 05; fever in Pt 06), which are very common in school among children. All of the infections responded to antibiotic treatment and did not require hospitalization.
The increased ratio of fungal skin infections in Pt 01 and Pt 03 (Pityriasis versicolor in both) and semisolid stools with positivity for Candida in Pt 01 was presumably related to immunosuppressive treatment with tacrolimus (Enderby et al, 2015) and to the lack of antifungal prophylaxis in these patients, that has to be considered crucial for future trials requiring the use of calcineurin inhibitors. These infections responded to a short-time local antifungal treatment.
The positivity for Helicobacter Pylori in Pt 05 is related to a family-spread infection that is very common among Arabics (Masoodi et al, 2015).
Overall, the safety profile of the immunosuppressive regimen, with regular monitoring of tacrolimus levels in blood, was considered good in our series of treated patients. Importantly, all of our patients were able to attend school while on immunosuppressive treatment.

Appendix Table S3: DNA chimerism in DMD patients

	**Biceps brachii**	**Tibialis anterior**
Pt01	0.07% (0.04-0.11)	0.00% (0.00-0.00)
Pt02	0.43% (0.00-0.69)	0.17% (0.06-0.53)
Pt03	0.07% (0.00-0.14)	0.14% (0.00-0.29)
	**Vastus lateralis**	**Gastrocnemius**
Pt05	0.04% (0.04-0.04)	0.13% (0.13-0.13)
Pt06	0.08% (0.04-0.11)	0.04% (0.00-0.04)

Appendix Table S4: MAB dosage

	I inf. n. x 10^8	II inf. n. x 10^8	III inf. n. x 10^8	IV inf. n. x 10^8	V inf. n. x 10^8	VI inf. n. x 10^8	Total n. x 10^8
Pt 01							
**LL** target	1.7	3.33	3.75	3.75			12.5
Infused dose	**1.57** (LLL)	**3.2** (LLL)	**2.1** (RLL)	**1.49** (RLL)			**8.36**
**UL** target			6.25	6.25			12.5
Infused dose			0	**2.98**			**2.98**
Pt 02							
**LL** target	0.83	1.67	1.75	1.75	3.2		9.2
Infused dose	**0.83** (RLL)	**1.67** (RLL)	**1.8** (LLL)	**1** (LLL)	**3.07**		**8.37**
**UL** target			3	3			6
Infused dose			**3**	**3**			**6**
Pt 03							
**LL** target	1.21	2.43	2.79	2.79			9.22
Infused dose	**1.21** (LLL)	**2.31** (LLL)	**4.65** (RLL)	**0.49** (LLL)			**8.66**
**UL** target			4.61	4.61			9.22
Infused dose			0	**2.94**			**2.94**
Pt 05							
**LL** target	3.8	3.8	7.6	7.6	7.2	7.2	37.2
Infused dose	**3.48**	**3.45**	**7.6**	**7.6**	**7.2**	**6.6**	**35.93**
Pt 06							
**LL** target	4.5	4.5	9	9			27
Infused dose	4.39	3.45	8.21	9			**25.05**

Legend: The table shows MAB dosages for each patient, sub-divided for limbs. MAB dosages are expressed as number of cells (n) per 10^8. LL: Lower Limbs; UL: Upper Limbs; RLL: Right Lower Limbs; LLS: Left Lower Limbs; unless specified, the total dose was equally divided in both limbs.

MAB infusions in first three patients (Pt01, Pt02, Pt03):
Target dose: $50x10^6$/kg ($0.5x10^8$/kg).
I Infusion: $3.33x10^6$ /kg
II Infusion: $6.67x10^6$/kg
III and IV infusion: $20x10^6$/kg
Pt01 (basal weight: 50 kg): total target dose: $25x10^8$ ($0.5x10^8$/kg);
Total infused dose: $11.34x10^8$ ($0.23x10^8$/kg)
Pt02 (basal weight: 25 kg): total target dose: $12x10^8$ ($0.5x10^8$/kg);
Total infused dose: $11.3x10^8$ ($0.45x10^8$/kg) + additional infusion: 14.37 ($0.57x10^8$/kg)
Pt03 (basal weight: 37 kg): total target dose: 18•$44x10^8$ (0•$5x10^8$/kg)
Total infused dose: $11.6x10^8$ ($0.31x10^8$/kg)

MAB Infusion in last two patients (Pt05, Pt06):
Target dose: $60x10^6$/kg ($0.6x10^8$/kg).
I and II infusion: $10x10^6$ /kg
III and IV infusion: $20x10^6$/kg
Pt05 (basal weight: 38 kg): total target dose: $22.8x10^8$ ($0.6x10^8$/kg);
Total infused dose: $22.13x10^8$ ($0.58x10^8$/kg) + Additional Infusion: 35.93 ($0.97x10^8$/kg)
Pt06 (basal weight: 46 kg): dose target total: $27x10^8$ ($0.6x10^8$/kg);
Total infused dose: $25.05x10^8$ ($0.54x10^8$/kg)

APPENDIX FIGURE LEGENDS

Appendix Figure S1: scheme of treatment in Duchenne patients. Pt 01, 02, 03, 05 and 06 underwent MAB infusion treatment from T0, and then every 2 months (blue arrow) until T6. Muscle biopsy was performed eight months after the first infusion (T8; red arrow) and only for Pt 05 and Pt 06 one month before the onset of the infusion (red arrow with asterisk). Time points of MRI muscle examination (violet arrow) and functional and quantitative muscle force measures (green arrow) are reported. Before MAB infusion, all the patients underwent at least one year of observational study with functional and quantitative muscle force measures.

Appendix Figure S2: Quantitative immune-fluorescence analysis of dystrophin expression.
(A) Expression of dystrophin by anti-dys2 antibody in pre and post-treated muscles of Pt01, Pt05 and Pt06, which was "quantified" as relative to control muscle (from age-matched control patients in 40 dystrophin-positive muscle fibers) and normalized to β-spectrin expression, as previously reported (Arechavala-Gomeza et al, 2010). Only in post-treated samples of Pt05 we observed few fibers with dystrophin-positive intensity within, or close to, the range of normal controls.
(B) Expression of dystrophin by anti-mandys106 antibody in pre and post-treated muscles of Pt01, Pt05 and Pt06, which was "quantified" as relative to control muscle (from age-matched control patients in 40 dystrophin-positive muscle fibers) and normalized to β-spectrin expression, as previously reported (Arechavala-Gomeza et al, 2010). Only in post-treated samples of Pt06 we observed few fibers with higher intensity of staining as compare to pre-treatment.

Appendix Figure S3: Dystrophin 6283 C>T mutation detection in Pt 05.
(A) Sanger sequencing of healthy control, Pt 05 pre- and post-treatment skeletal muscle biopsy cDNA samples and donor genomic DNA sample. Mutation 6283C>T is underlined. In both pre- and post-treatment Pt 05 samples T allele (leading to premature STOP-codon) can only be detected.
(B) Tetra-primer ARMS PCR was carried out to characterize the 6283 C>T mutation. In a single PCR reaction outside primers (F1 and R1) were combined with allele specific inner primers R(T) and F(C). The inner primers were designed to harbour mismatch at position -2 to enhance specificity. T allele amplification leads to 263 bp product size and C allele to 237 bp PCR product. Allele specific internal primers help to minimize the risk of potential allele-specific bias in detecting polymorphisms. The accompanying gel demonstrates that in both pre- and post-treatment Pt 05 samples tetra-primer ARMS PCR detects primarily T allele and in healthy control C allele. The alternative allele can be attributed to background signal.

Appendix Figure S4: Representative percentile of MRI quantitative parameters in transplanted patients compared to untreated patients.
(A) MVI of the thigh biceps.
(B) SIR of thigh flexor muscles.
(C) MVI of the semitendineous muscles.
(D) MVI of the soleus muscles.
Pt05 is the only patient showing changes in the trend after treatment. Empty dots correspond to pre-treatment measures. Orange dots correspond to post-transplantation measurement. The black line corresponds to the median, while grey dotted lines to

75th, 90th and 95th percentile.

Appendix Figure S5: Kin Com measures plotted against patients'age.

Pt01 Knee Extension: strength was set on low values and remained quite stable until the age of 15. After this time point, the patient lost the ability to perform an active knee extension movement. Knee Flexion: strength was set at the same level of knee extension and it was constant during time until a drop at the age of 15. Elbow Flexion/Extension: strength values of upper limbs were higher than lower limbs, especially in elbow extension. Elbow flexion decreased more rapidly than elbow extension until they reached almost the same values around the age of 14.5.

Pt 02 Knee Extension: a remarkable drop of strength happened just before the onset of MAB infusion and a second drop occurred around the age of 11 (2.5 years after the first MAB infusion) causing the inability of performing an active knee extension movement. Knee Flexion: the strength values were lower at baseline as compared to knee extension (as expected in physiological conditions). The strength gradually reduced until loss of active movement, around the age of 11. Elbow Flexion/Extension: the strength was maintained relatively constant until the age of 9. Although a momentary increase in strength occurred after the age of 9, it was followed by a severe decrease indicating the inability of performing elbow extension/flexion against gravity after the age of 10.

Pt 03 Knee Extension: the strength was remarkable reduced before the onset of MAB infusion. After MAB infusion, the strength was maintained constant. Knee Flexion: the strength decreased gradually over time before the age of 9 and showed a quite constant pattern during the year of MAB infusion. The strength dropped rapidly when the patient reached the age of 11. The isometric performance always showed higher level of strength than the isokinetic performance. Elbow Flexion/Extension: after a drop of strength before MAB infusion, the pattern of strength was constant over time.

Pt 05 Knee Extension: the strength decreased just before the onset of MAB infusion, and then it was stable until the age of 10 when again mildly decreased. Knee Flexion: the strength mildly decreased before the onset of MAB injections; then it was stable until the age of 10 when showed a drop followed by a rapid increase in strength. Except for one measurement between 10 and 11 years old, isometric contraction always showed higher levels of strength than isokinetic contraction. Elbow Flexion/Extension: strength increased until the age of 9, then, dropped and stabilized at a constant low level.

Pt 06 Knee Extension: the strength progressively decreased before the onset of MAB infusion; then it was stable until the age of 13 when a second drop occurred. Knee Flexion: the strength was much higher as compared to the other DMD patients, and showed increased values of isometric contraction after the age of 10.5. Isometric values were always higher than the isokinetic ones, which were quite stable until a light drop around the age of 13. Elbow Flexion/Extension: After an increase and then a light decrease between 10 and 11 years old, upper limbs showed stable levels of strength.

Legend: arrows represent time points of MAB infusion; red arrow means that MABs were infused in that corresponding limb; grey arrow means that MABs were not infused in that corresponding limb.

Appendix Figure S6: Kin Com measures of Pt05

Data are plotted against years before and after the onset of MAB infusion. A decrease in the slope of change is present after MAB infusion.

KE = knee extension; KF = knee flexion; EE: elbow extension; EF = elbow flexion.

Appendix Figure S7: Phenotype and absolute counts of circulating lymphocytes, dystrophin-specific humoral responses and lytic activity of Pt03 against donor cells.

(A-F) Immune cell counts were measured at different time-points, namely before the beginning of treatment (PRE), prior to each infusion and 2 months after the fourth infusion. Results are expressed as absolute counts of $CD3^{+}$ (A), $CD3^{+}CD4^{+}$ (B) or $CD3^{+}CD8^{+}$ (C) T cells, $CD16^{+}/CD56^{+}$ NK cells (D) and $CD19^{+}$ B cells (E) per μl. Grey dotted lines indicate the reference range calculated on a cohort of pediatric healthy donors. Mean and SD are shown.

(F): The differentiation phenotype of T cells was assessed by FACS. The relative proportion of naïve T cells (T_{Na}, $CD45RA^{+}CD62L^{+}$), central memory T cells (T_{CM}, $CD45RA^{-}CD62L^{+}$), effector memory T cells (T_{EM}, $CD45RA^{-}CD62L^{-}$) and effector T cells expressing CD45RA ($T_{EM}RA$, $CD45RA^{+}CD62L^{-}$) was calculated by gating on $CD3^{+}$cells. Both age-matched healthy donors (CTR) and age-matched DMD patients under steroid treatment (CTR DMD) were used as controls.

(G) Dystrophin specific humoral responses were assessed by western blot analysis. Proteins extracted from a human muscle biopsy harvested from a non-dystrophic donor, were incubated with patients' sera at two different time points: before the beginning of treatment (PRE) and 2 month after the fourth infusion. Donor sera were used as healthy controls (DN) while the monoclonal antibody DYS-1 was used to identify the correct molecular weight of the dystrophin. Results for Pt01, Pt02, Pt03, Pt05 and Pt06 and for two representative donors are shown. As a positive control, the same sera were tested on membranes blotted with influenza virus proteins. A monoclonal antibody recognizing a conformational epitope of HA protein that is conserved in H1N1 and H2N2 subtypes was used to verify the integrity of influenza virus proteins. Results for two representative donors are shown.

(H) PBMC harvested from Pt03 two months after the last infusion (10m), were stimulated *in-vitro* with irradiated MAB isolated from the donor. Lytic activity of responder T lymphocytes was measured 14 days after the third round of stimulation against several ^{51}Cr labeled target cells obtained from the donor: MABs, IFN-γ treated MABs (γMAB), myotubes derived from MABs and the donor PHA line. Autologous PHA line was used as negative control.

Appendix Figure S8: Angiographic evaluation pre and post limb infusion.

(A) Preliminary angiography of the left lower limb, before MAB infusion, shows patency of popliteal and tibial arteries.

(B) After MAB infusion, angiography confirms patency of popliteal and tibial arteries.

(C) Preliminary angiography of the right upper limb, before MAB infusion, shows patency of the brachial, radial and ulnar arteries.

(D) After MAB infusion, angiography confirms patency of brachial, radial and ulnar arteries.

Appendix Figure S9: Donor MABs form myotubes in vitro.

Spontaneous differentiation of donor MABs obtained from the medicinal product before infusion. $2x10^{5}$ MABs were plated on low-growth factor matrigel coated 3.5

cm Petri dish, in proliferation medium (Megacell). After an O/N incubation at 37°C, 5% CO2, proliferation medium was replaced by differentiation medium (DMEM supplemented with 2% horse serum), and differentiation extended for 10 days. Cells were stained with anti myosin heavy chain antibody and Dapi. Bar = 30μm.

References

Arechavala-Gomeza V, Kinali M, Feng L, Brown SC, Sewry C, Morgan JE, Muntoni F (2010) Immunohistological intensity measurements as a tool to assess sarcolemma-associated protein expression. *Neuropathology and applied neurobiology* **36:** 265-274

Enderby C1, Keller CA 2015 An overview of immunosuppression in solid organ transplantation. *Am J Manag Care*. **21** (1) :s12-23.

Masoodi M, Talebi-Taher M, Tabatabaie K, Khaleghi S, Faghihi AH, Agah S, Asadi R (2015) Clarithromycin vs. Gemifloxacin in Quadruple Therapy Regimens for Empiric Primary Treatment of Helicobacter pylori Infection: A Randomized Clinical Trial. *Middle East J Dig Dis*. **7** (2) :88-93.

Appendix Figure S1

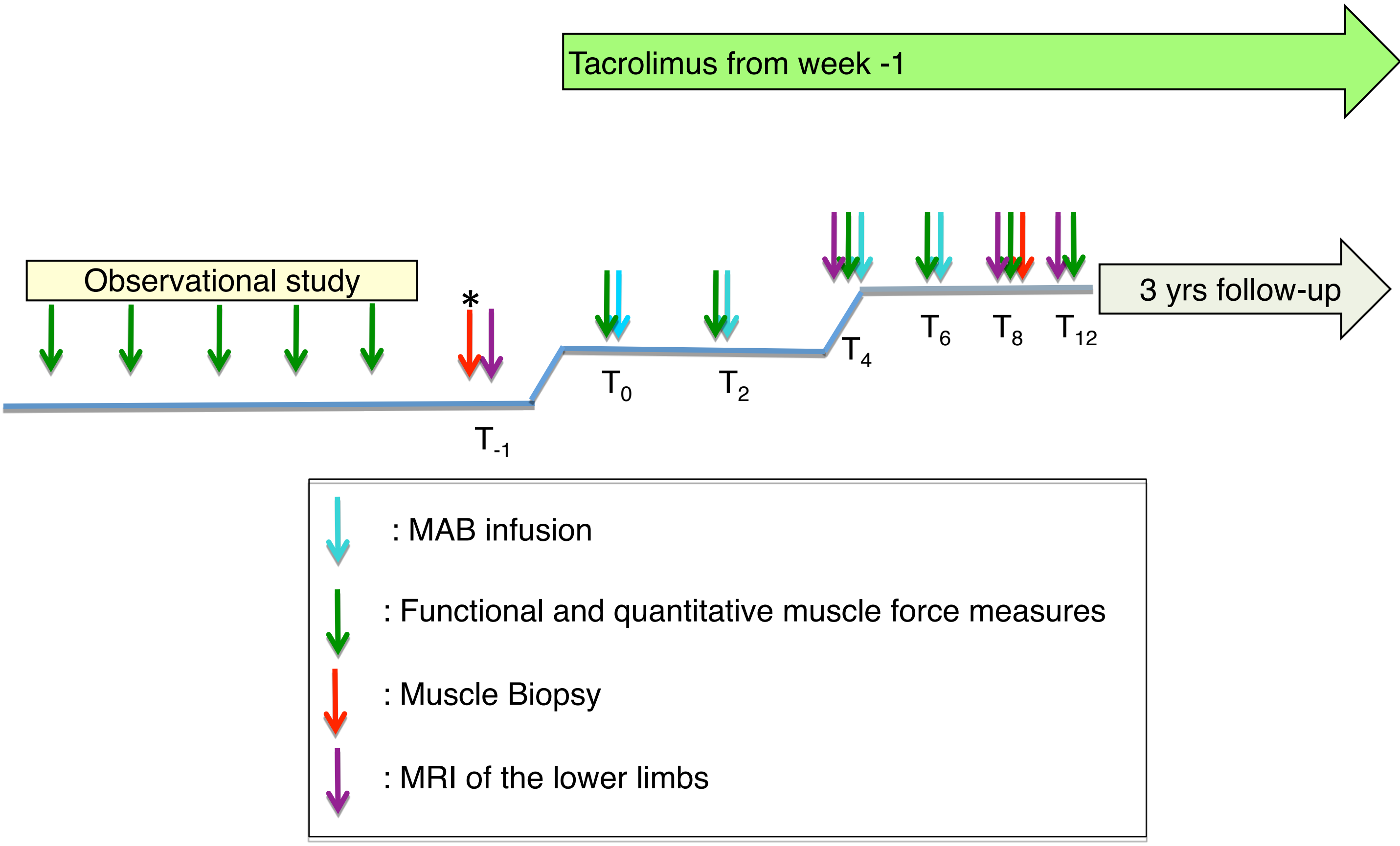

Appendix Figure S2

A

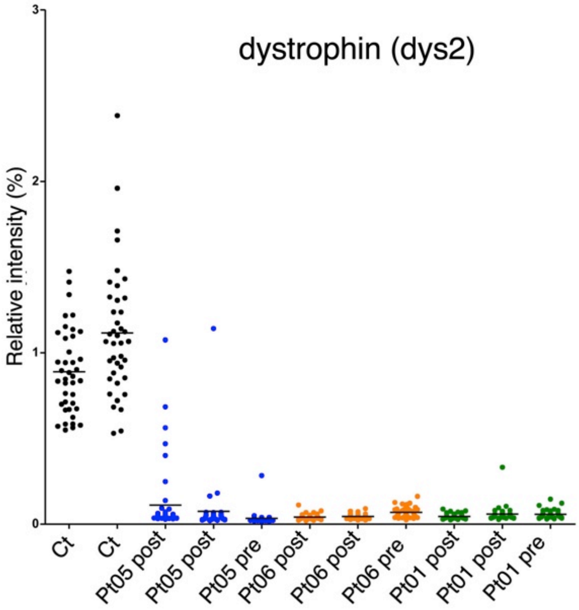

B

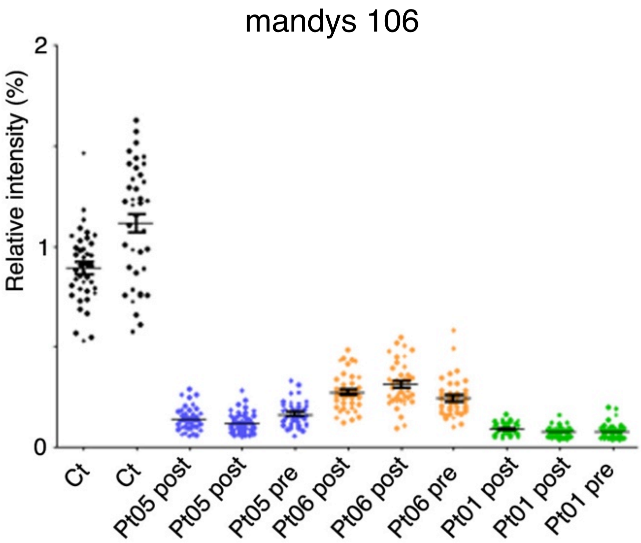

Appendix Figure S3

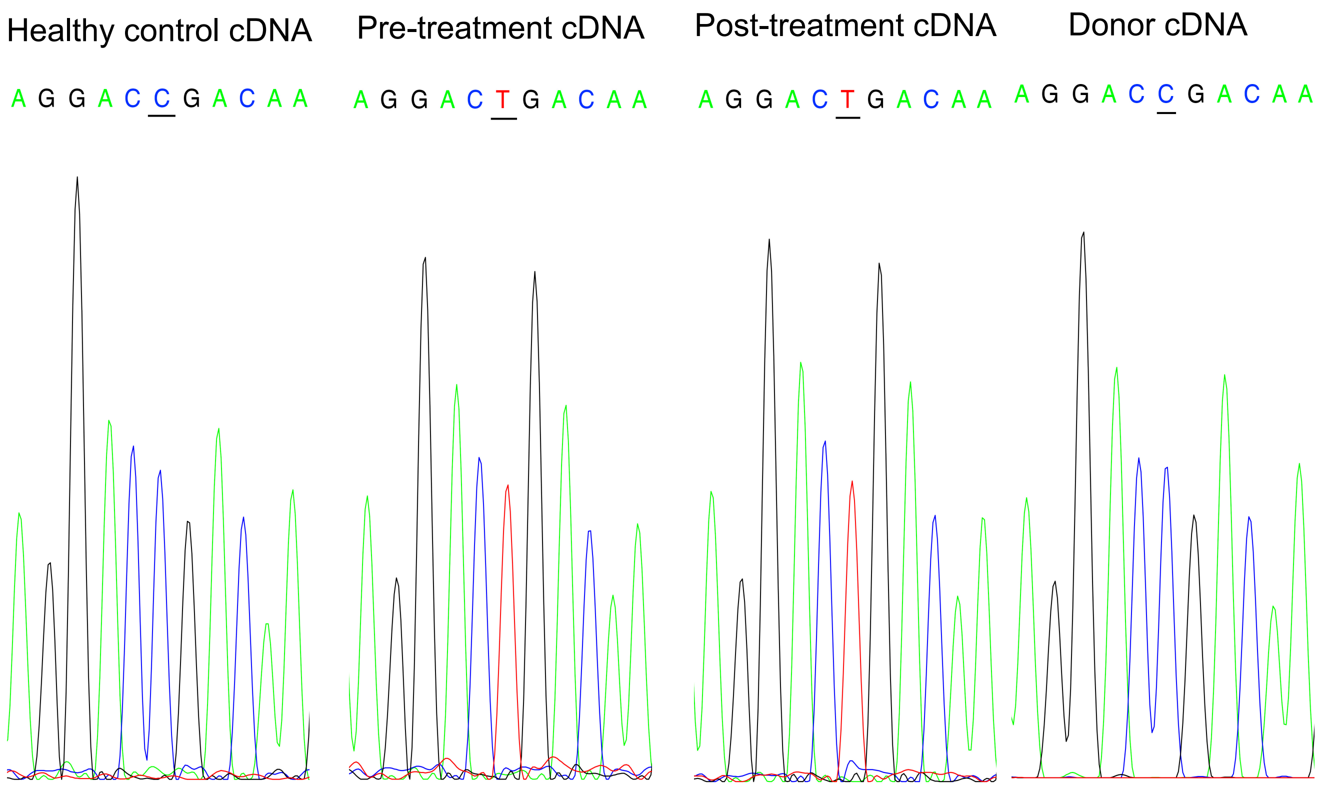

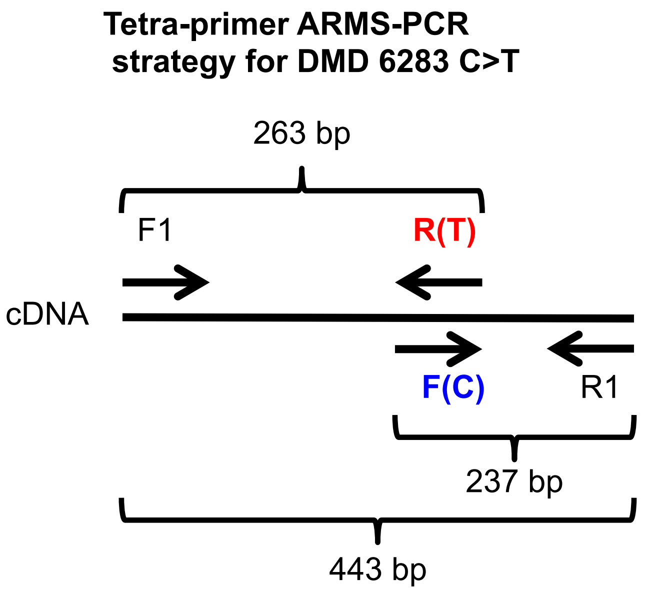

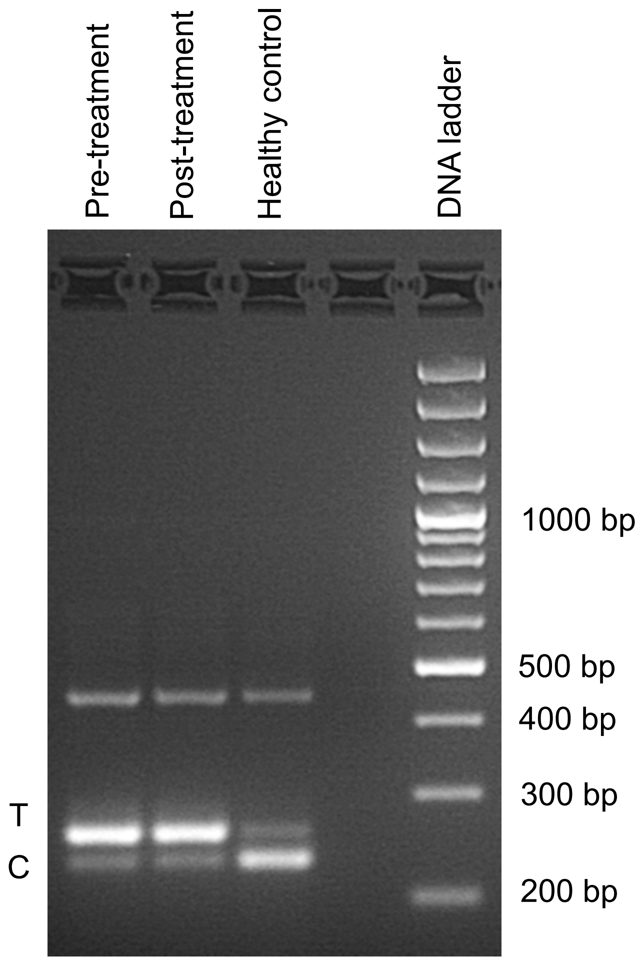

Appendix Figure S4

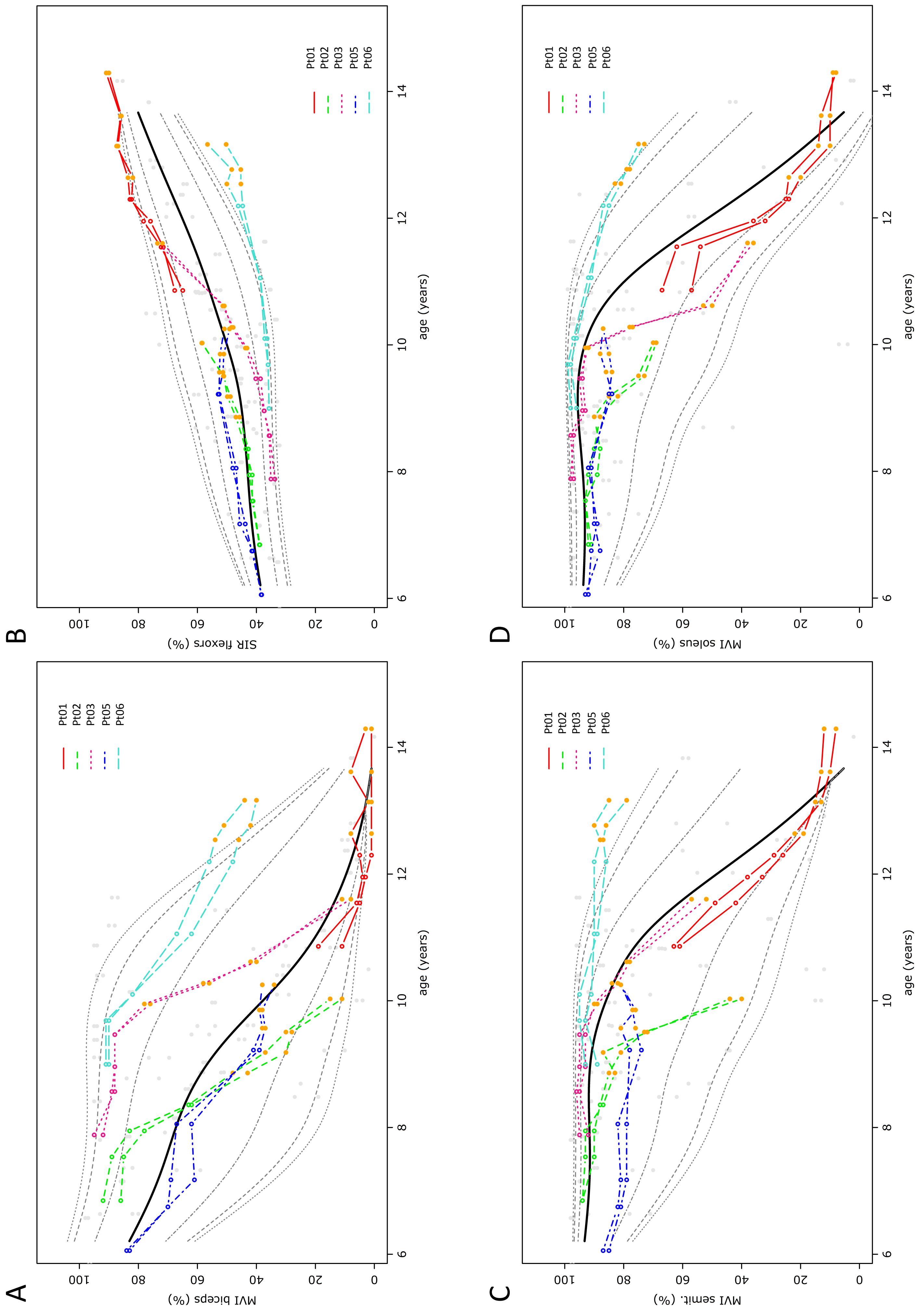

Appendix Figure S5

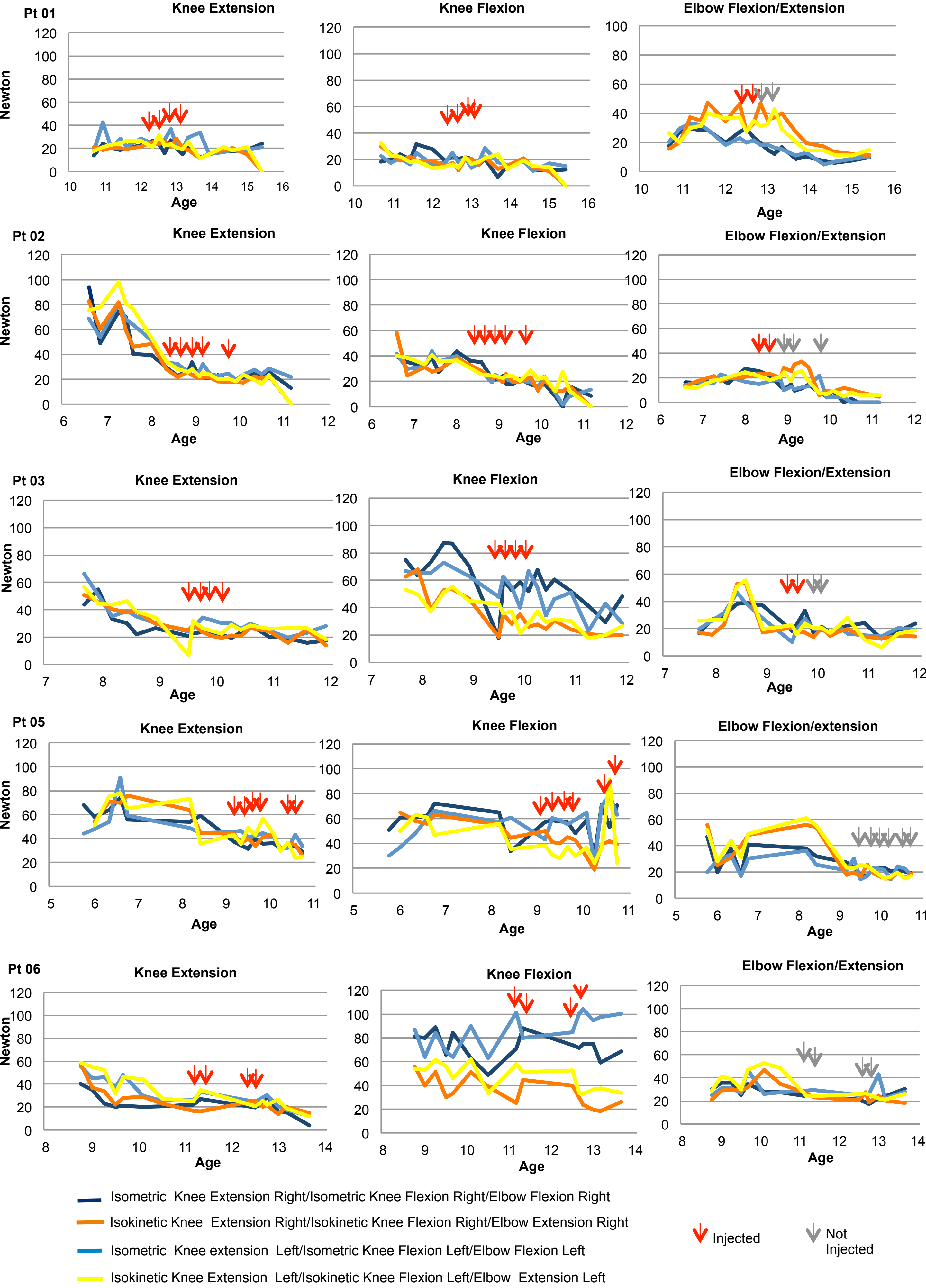

Appendix Figure S6

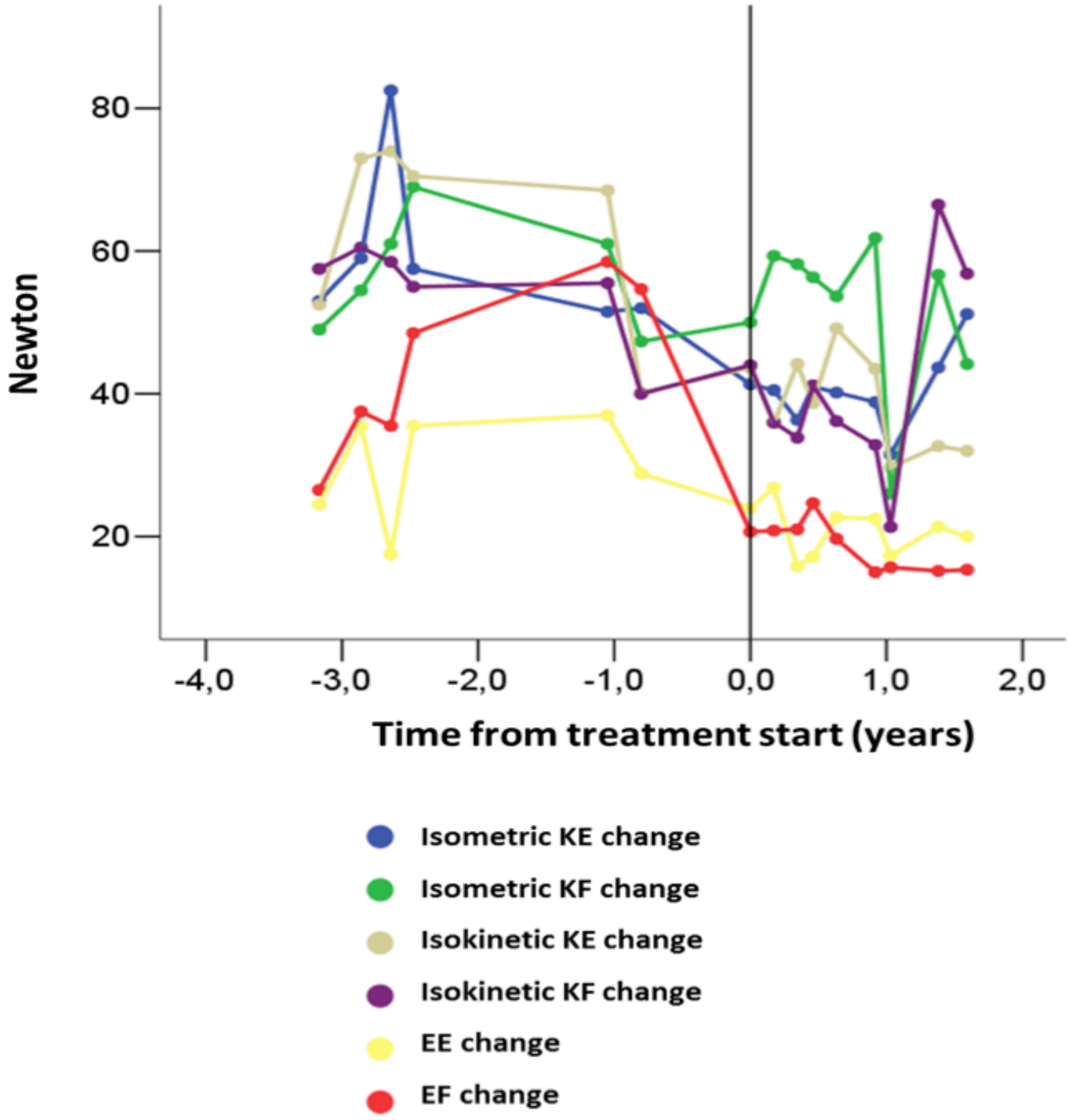

Appendix Figure S7

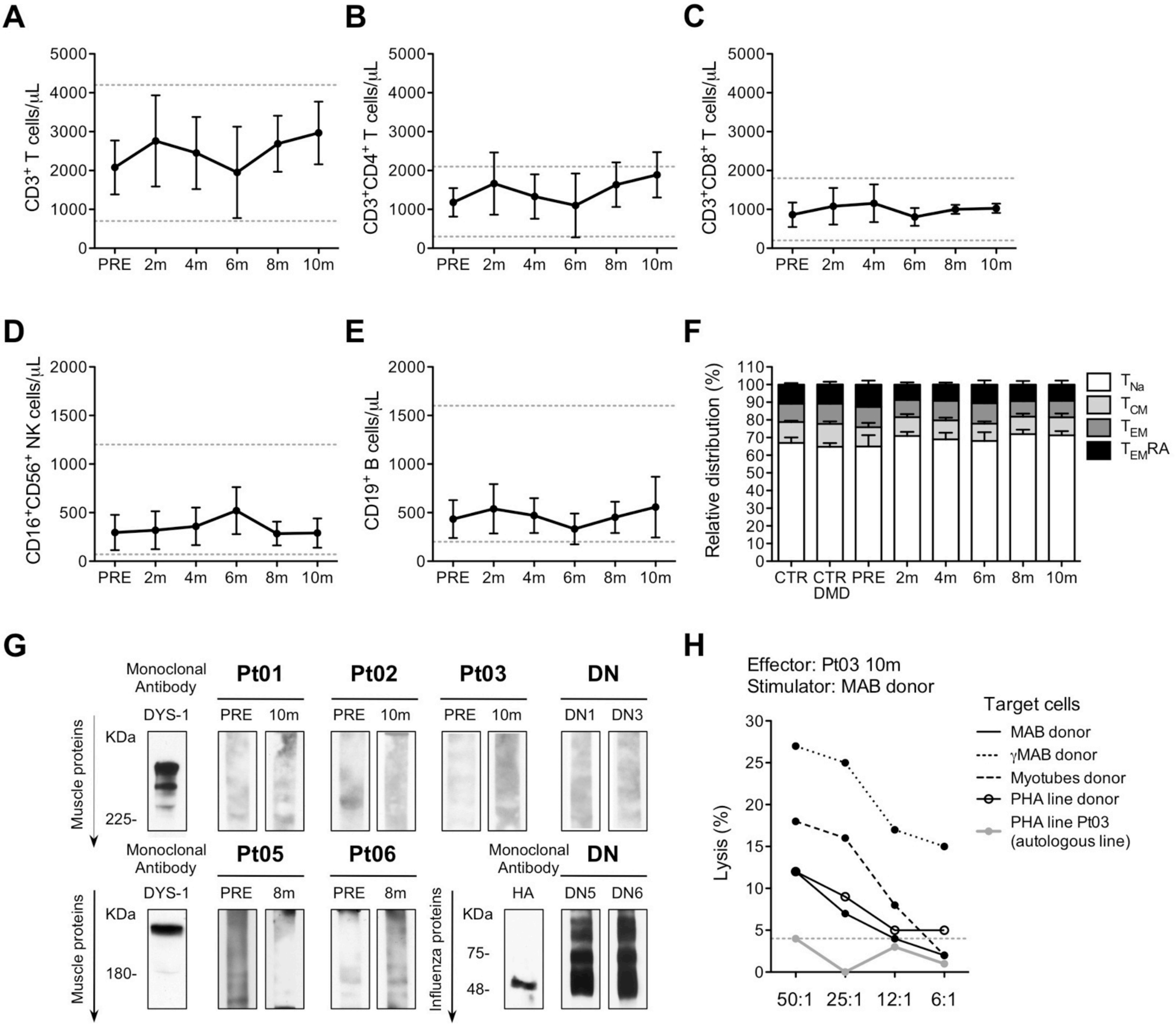

Appendix Figure S8

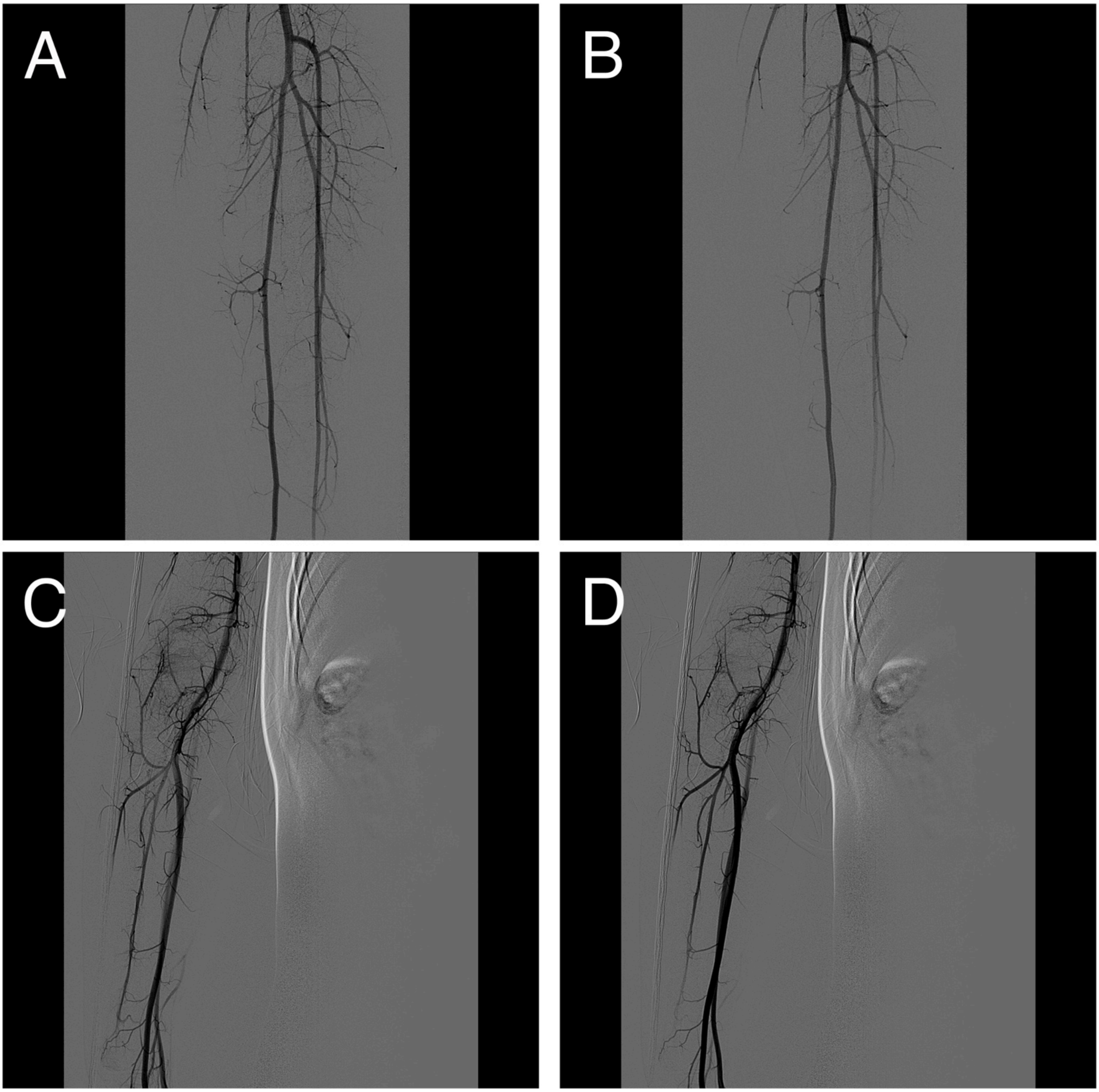

Appendix Figure S9

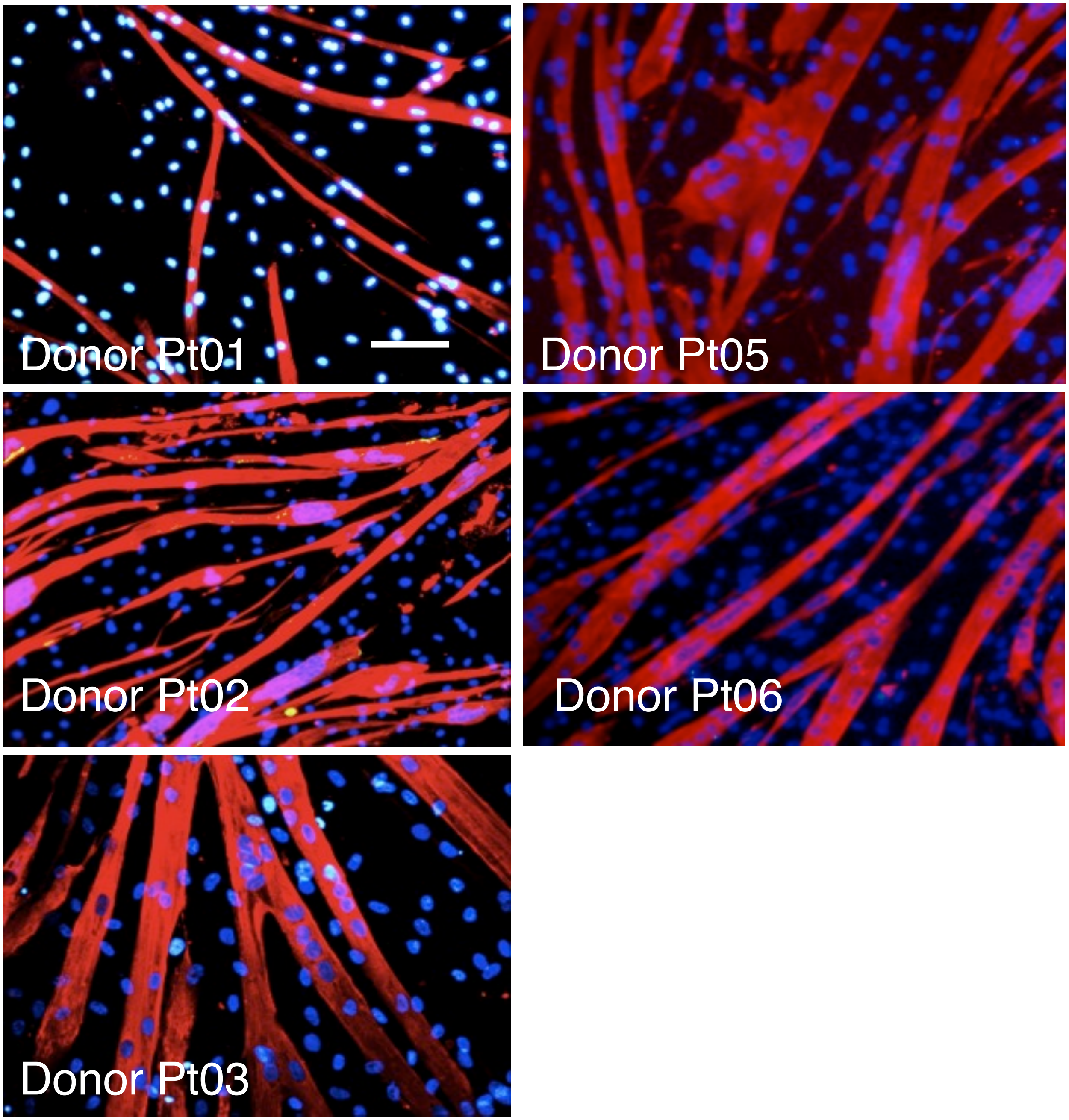

Supplement: Supplementary file 1 — Appendix [file EMMM-7-1513-s001.pdf]
