# Supplementary material for: Intra‐arterial transplantation of HLA‐matched donor mesoangioblasts in Duchenne muscular dystrophy
Source: EMBO Mol Med. 2015 Nov 5;7(12):1513–28. doi: 10.15252/emmm.201505636 (PMC4693504; doi:10.15252/emmm.201505636)

Pt01-DN1

Muscle proteins

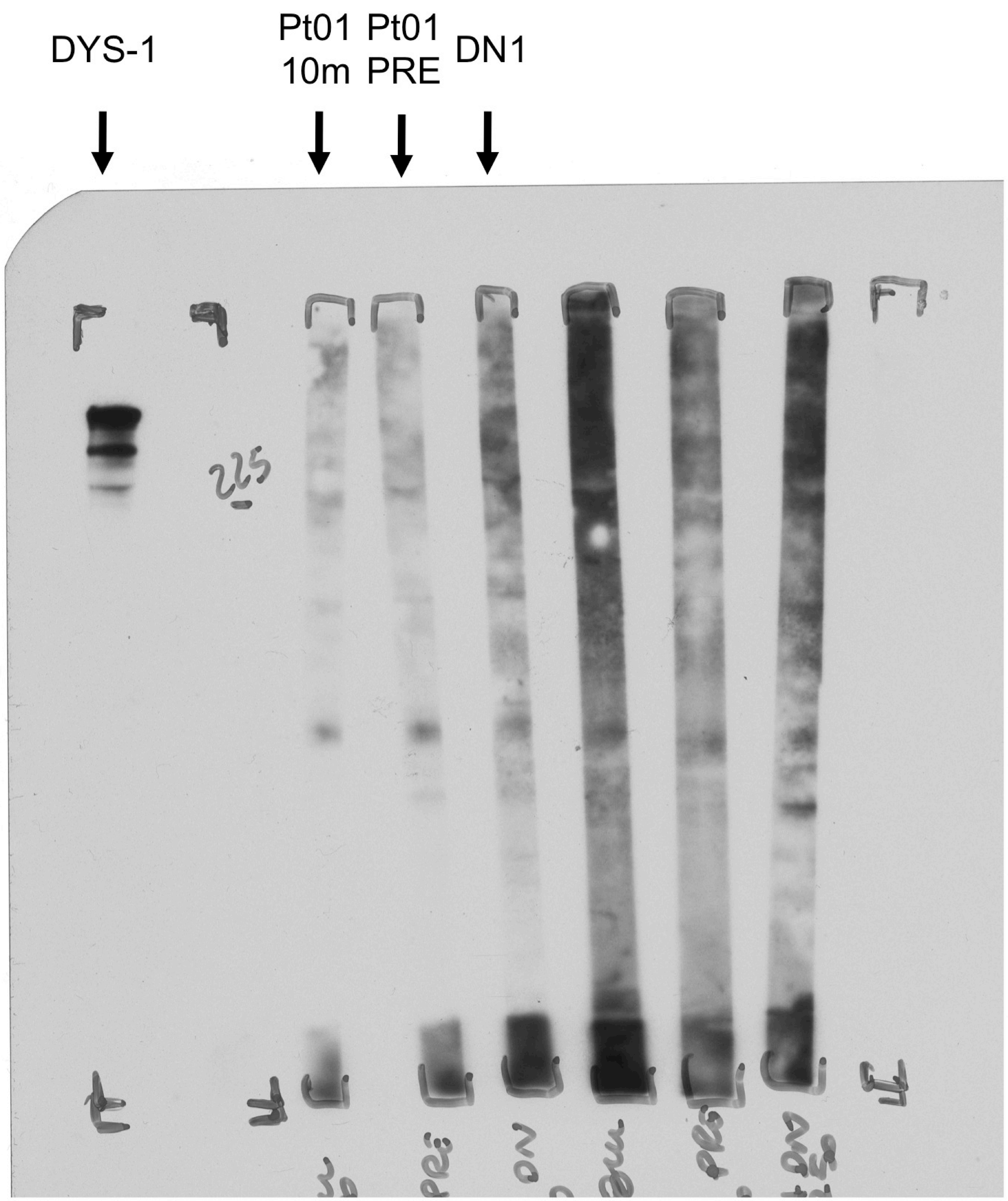

Exposure time: 5 minutes

Lane 1: DYS-1 monoclonal antibody

Lane 2: Pt01 10m

Lane 3: Pt01 PRE

Lane 4: DN1

Pt02

Muscle proteins

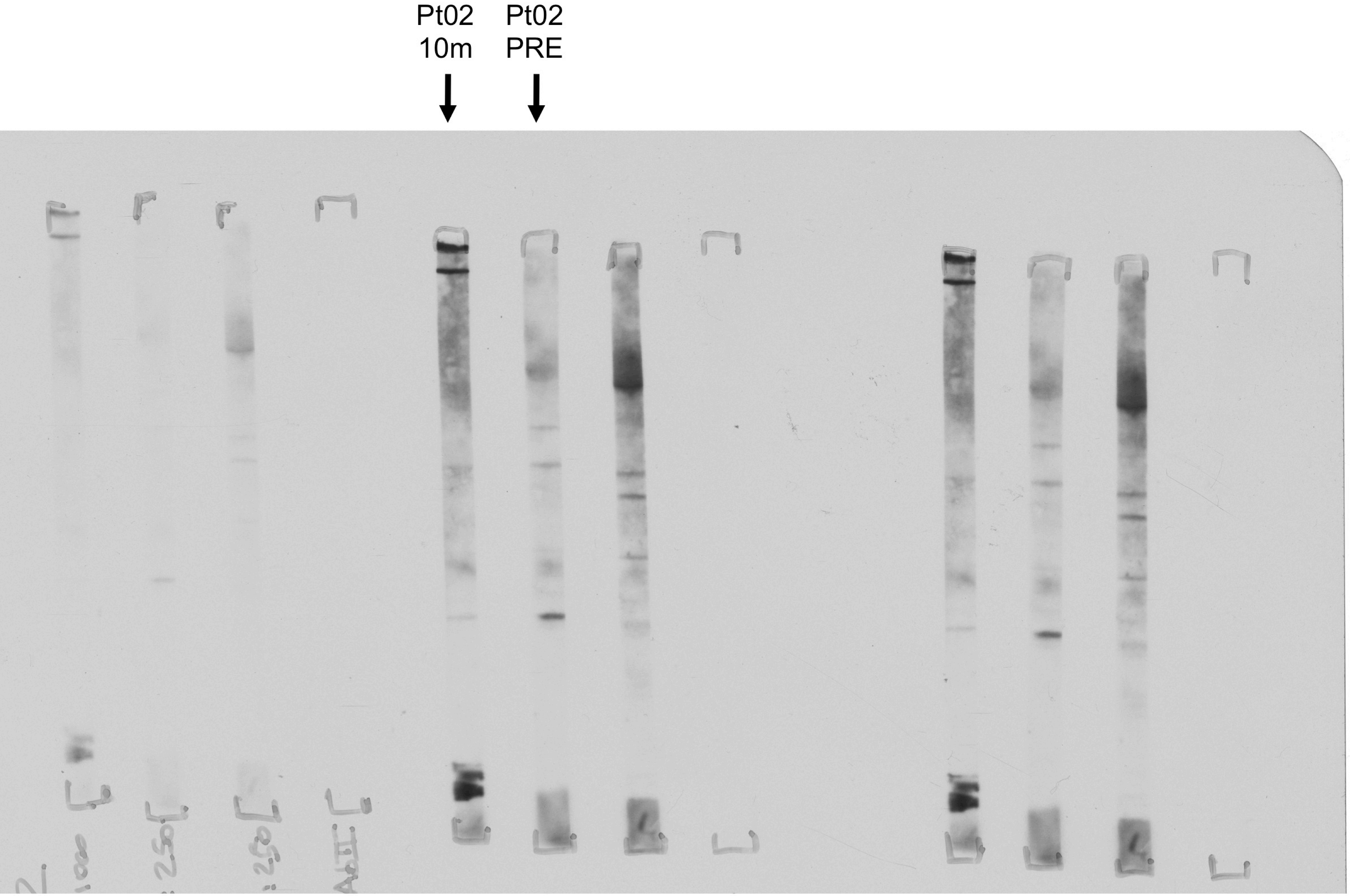

Exposure time:
5 minutes

Lane 5: Pt02 10m
Lane 6: Pt02 PRE

Pt03-DN3

Muscle proteins

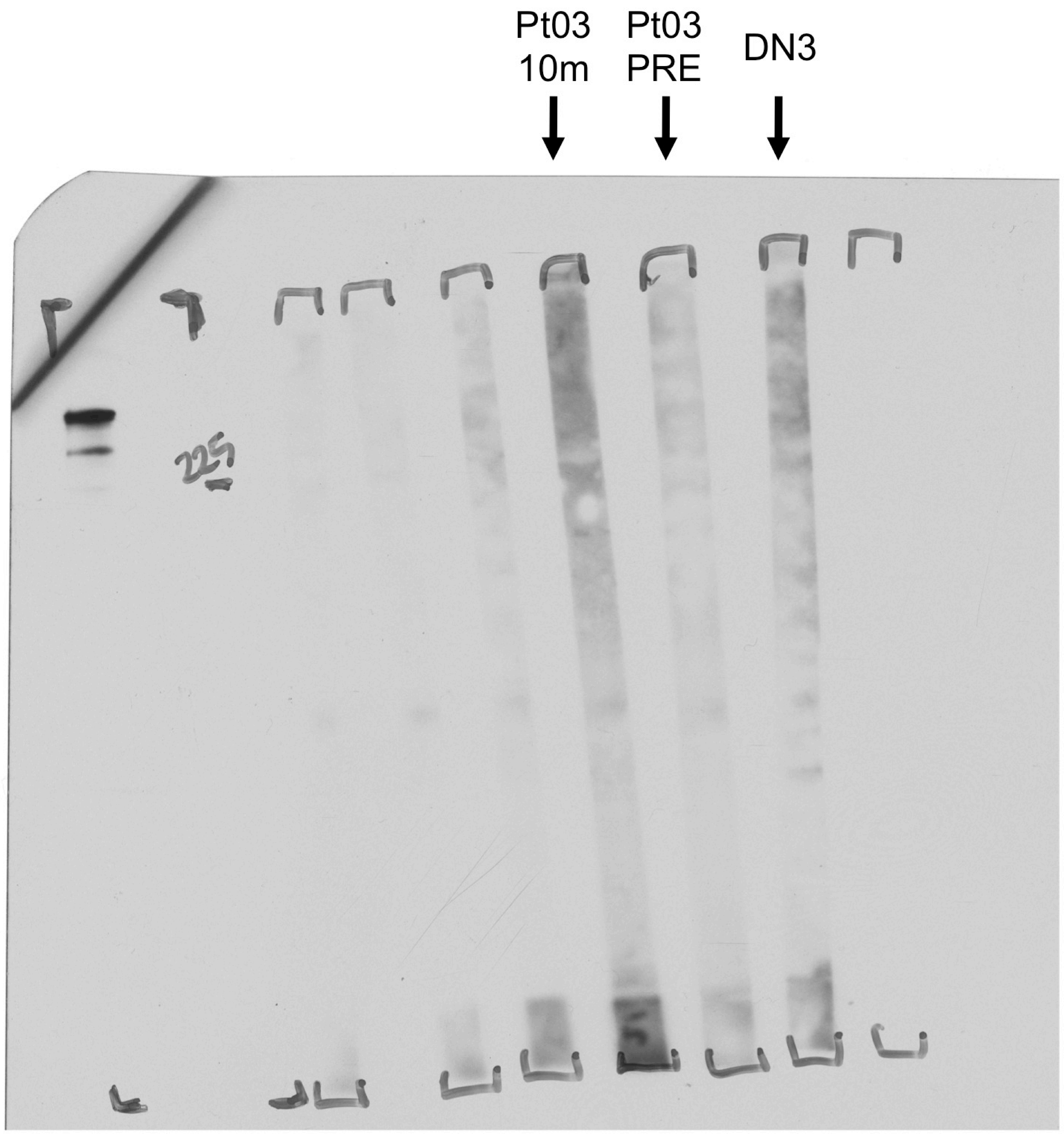

Exposure time: 30 seconds

Lane 5: Pt03 10m

Lane 6: Pt03 PRE

Lane 7: DN3

Pt05

Muscle proteins

Exposure time: 1 second

Lane 3: Pt05 PRE

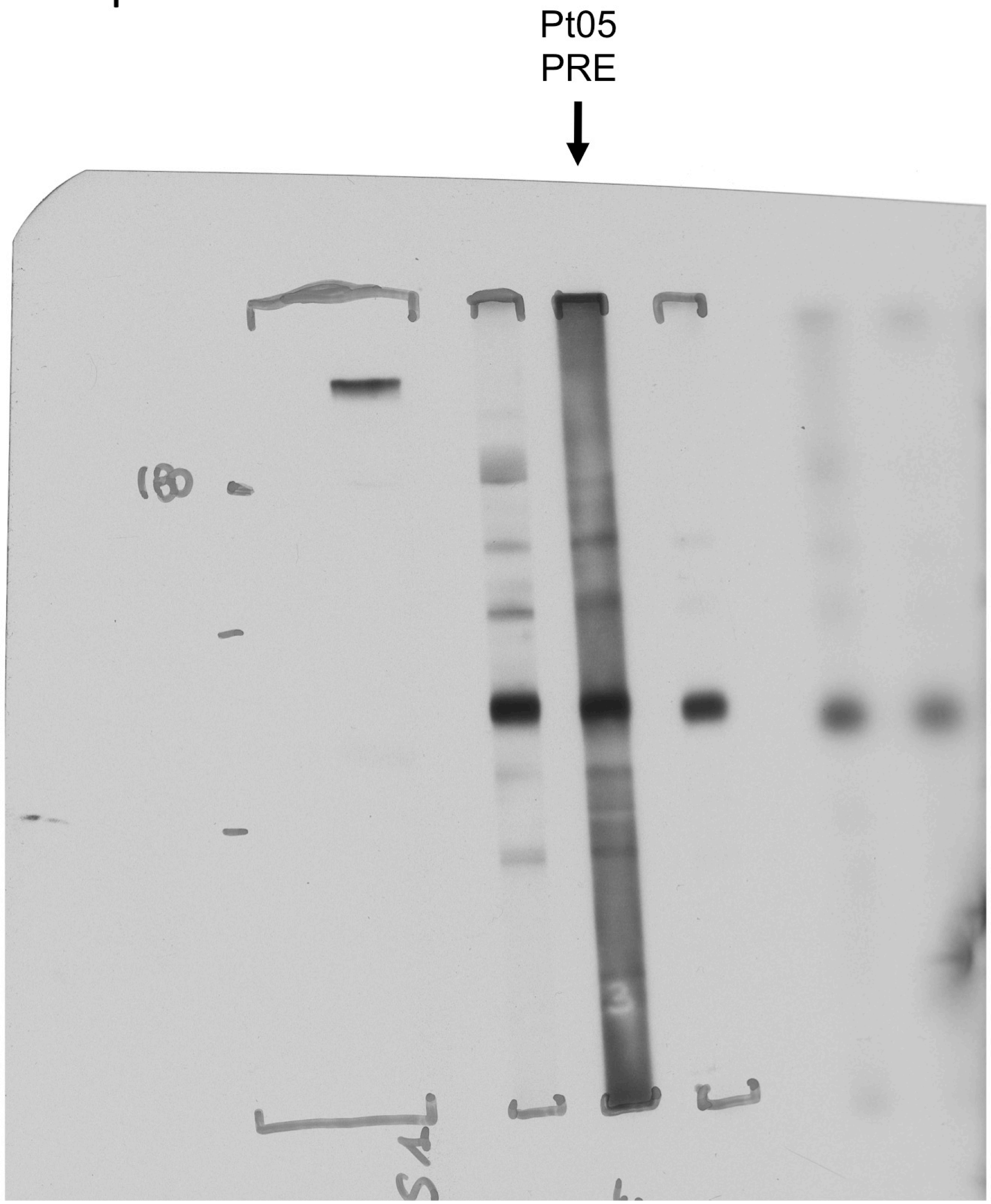

Pt05

Muscle proteins

Exposure time: 1 minute

Lane 4: Pt05 8m

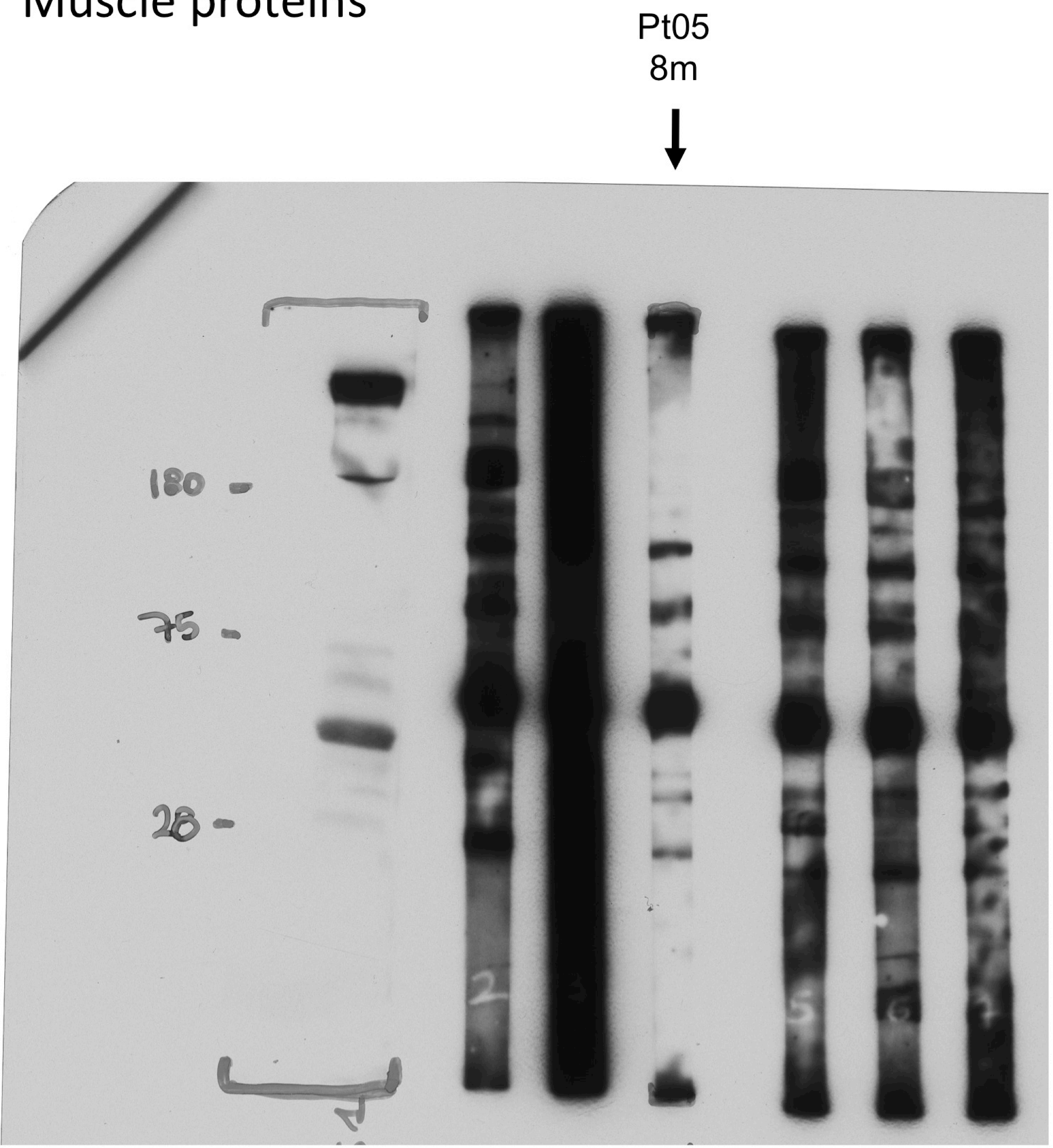

Pt06

Muscle proteins

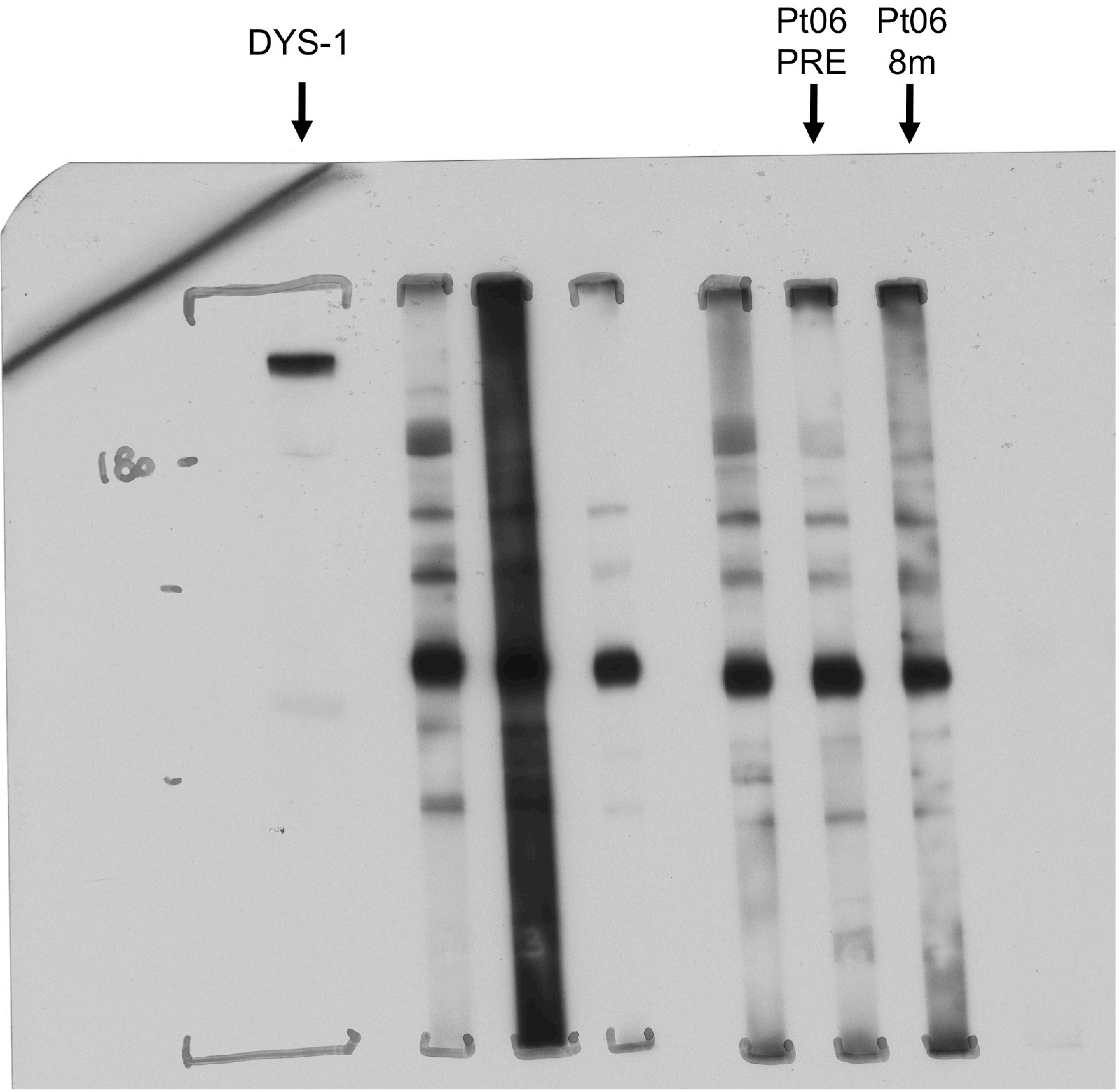

Exposure time: 2 seconds

Lane 1: DYS-1

Lane 6: Pt06 PRE

Lane 7: Pt06 8m

HA Positive control

Influenza proteins

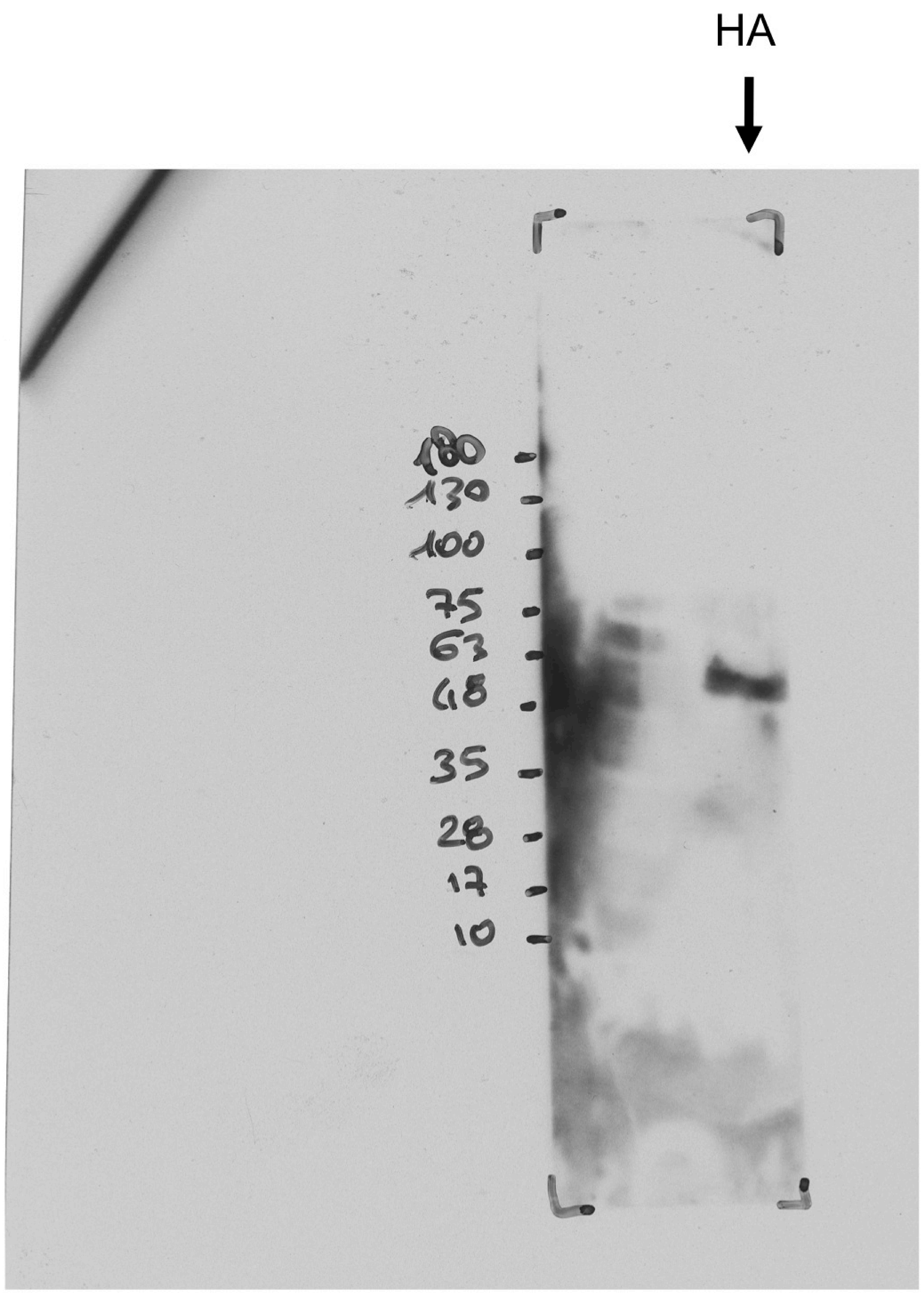

Exposure time: 10 minutes

Lane 1: HA monoclonal antibody

DN5-DN6

Influenza proteins

Exposure time: 30 seconds

Lane 2: DN5

Lane 5: DN6

DN5 DN6

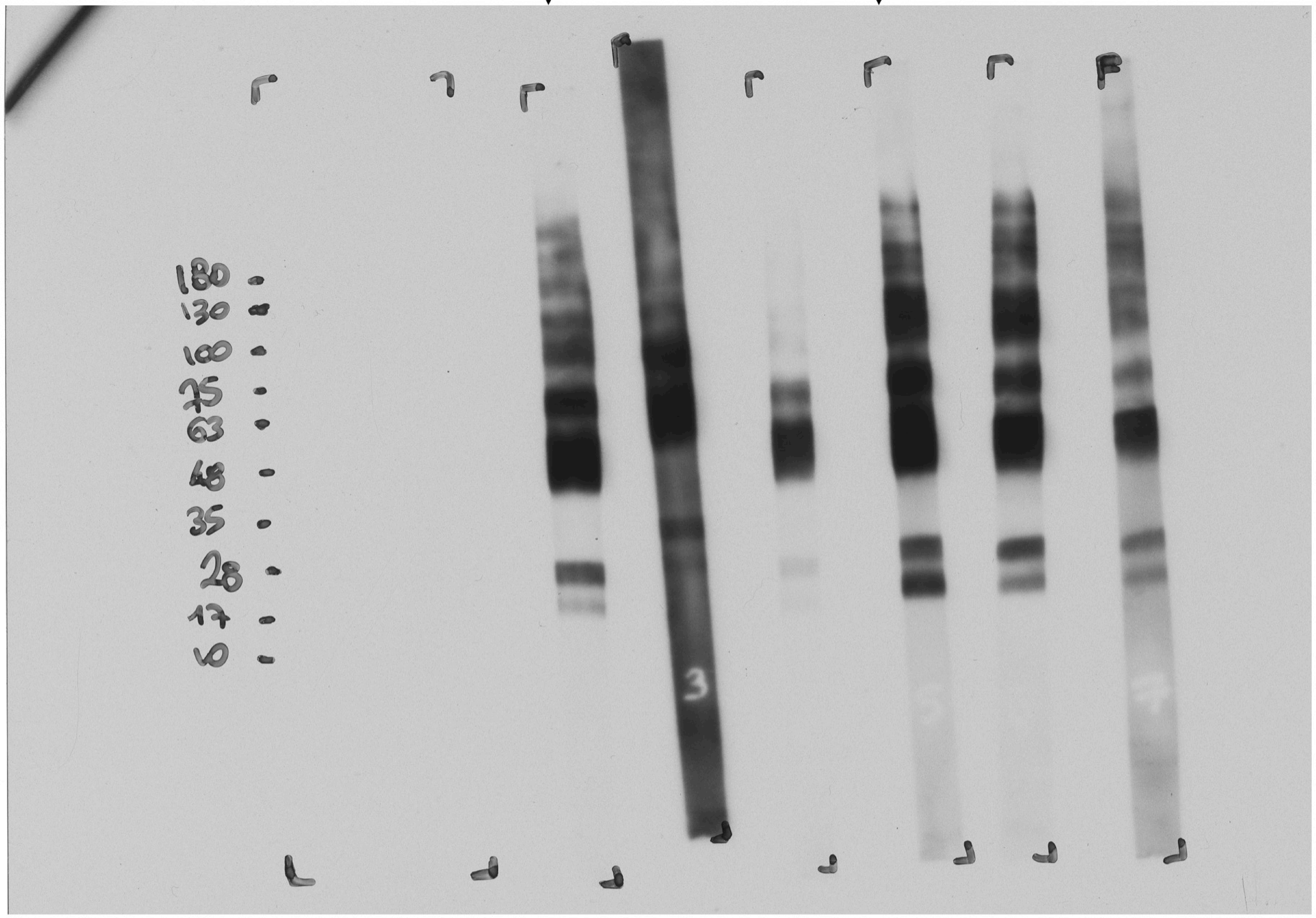

Supplement: Supplementary file 2 — Source Data for Appendix Figure S7 [file EMMM-7-1513-s003.zip › Source Data for Appendix/Source Data Appendix Fig S7.pdf]
