# Supplementary material for: Intra‐arterial transplantation of HLA‐matched donor mesoangioblasts in Duchenne muscular dystrophy
Source: EMBO Mol Med. 2015 Nov 5;7(12):1513–28. doi: 10.15252/emmm.201505636 (PMC4693504; doi:10.15252/emmm.201505636)

figure 3E myosin

Ct Pt01 Ct Pt01 Ct Pt01

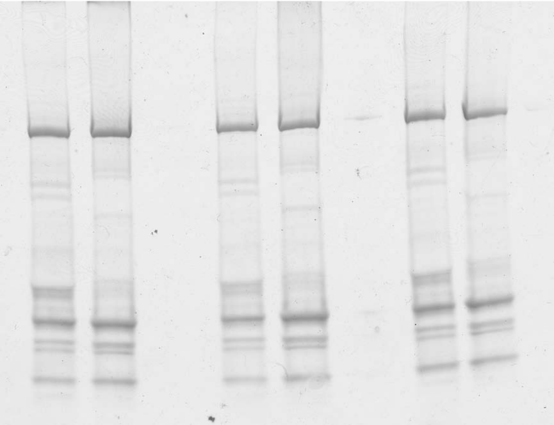

figure 3E

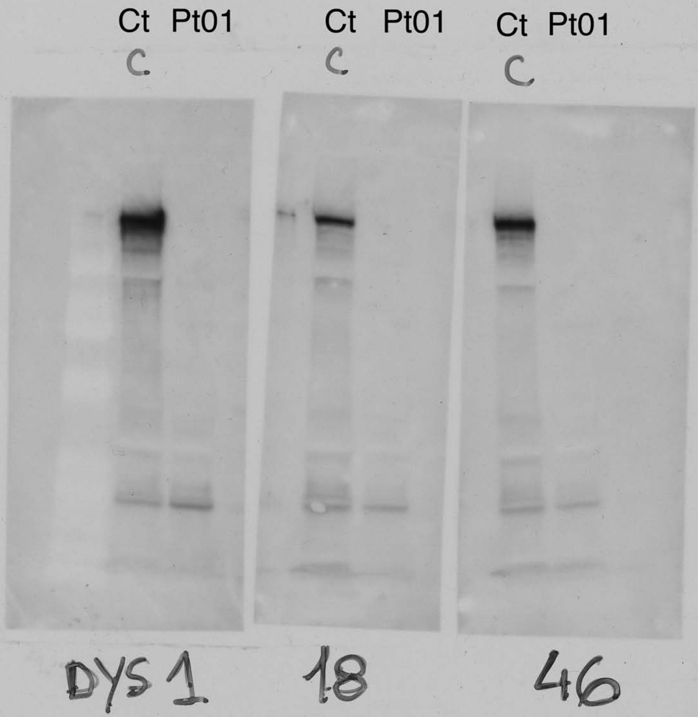

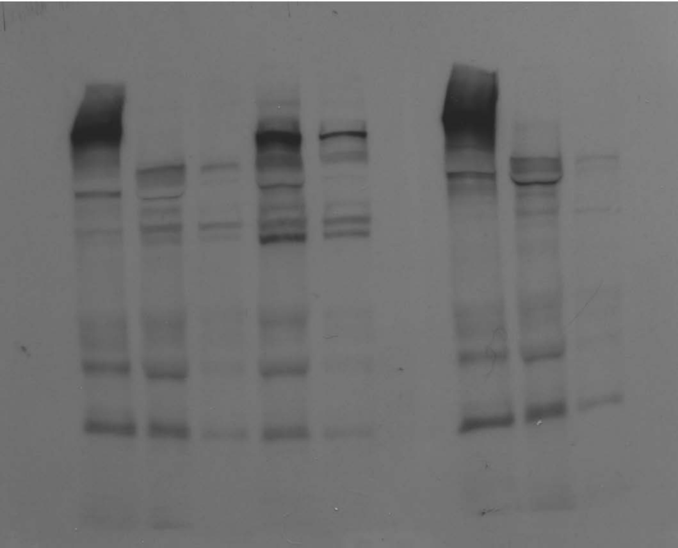

FIGURE 3F
mandys46

FIGURE 3F
myosin

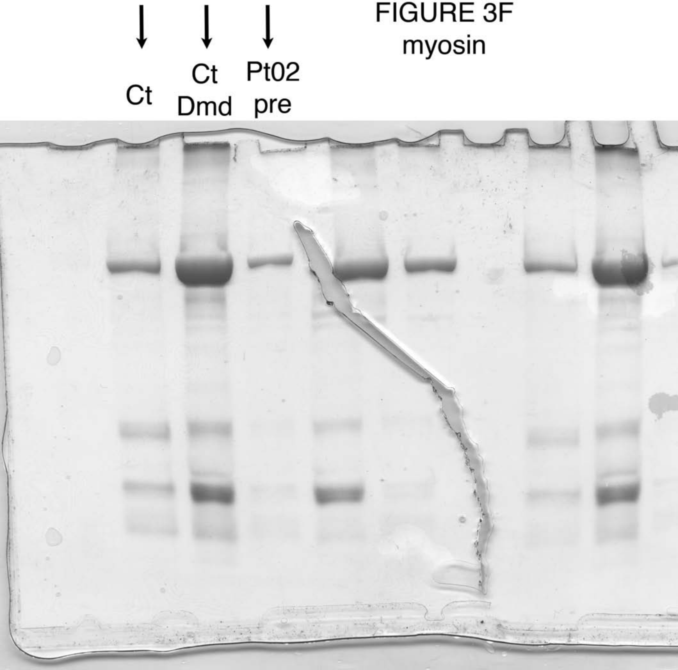

figure 3F
man46

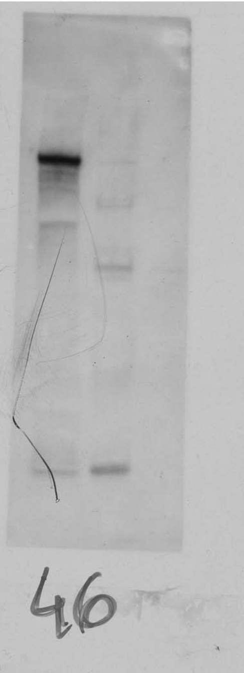

FIGURE 3F myosin

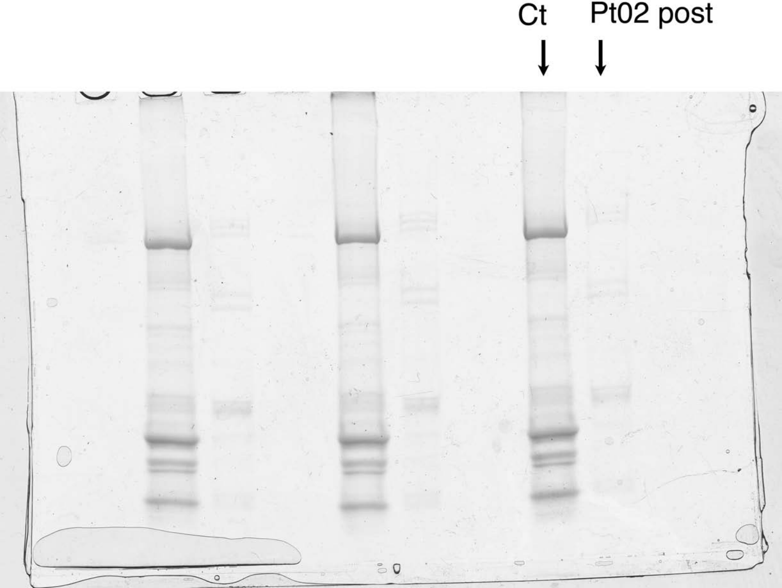

FIGURE 3F

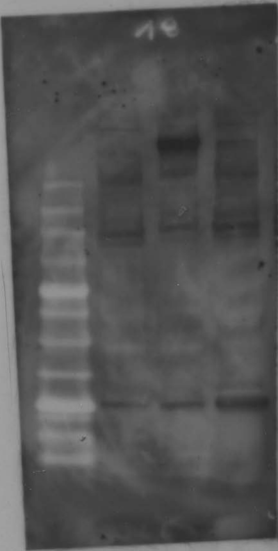

FIGURE 3F myosin

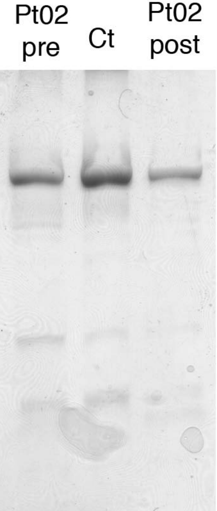

FIGURE 3G

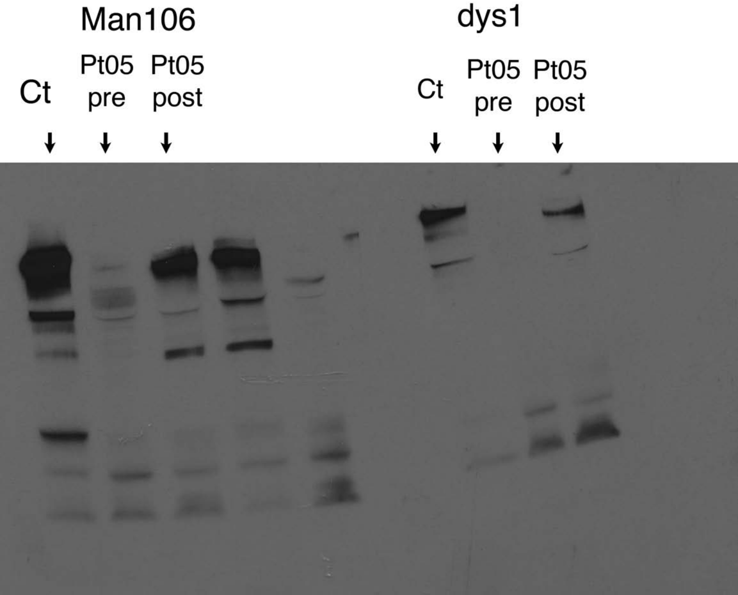

FIGURE 3G myosin

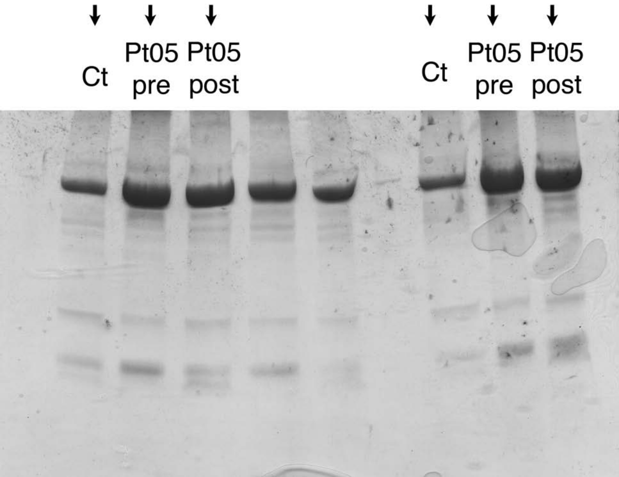

FIGURE 3H
man106

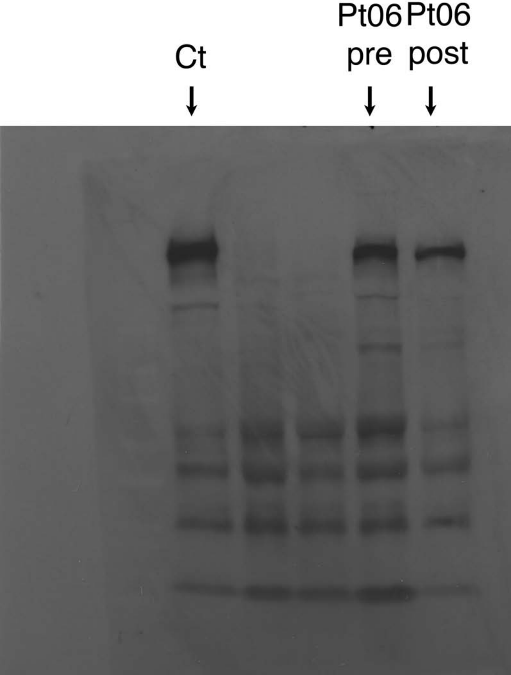

figure 3H

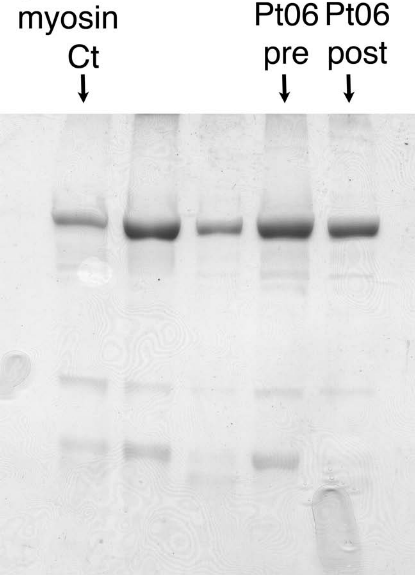

FIGURE 3H

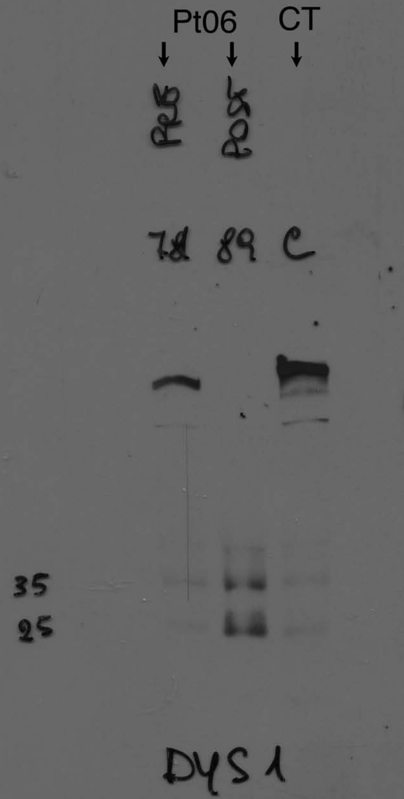

FIGURE 3H myosin

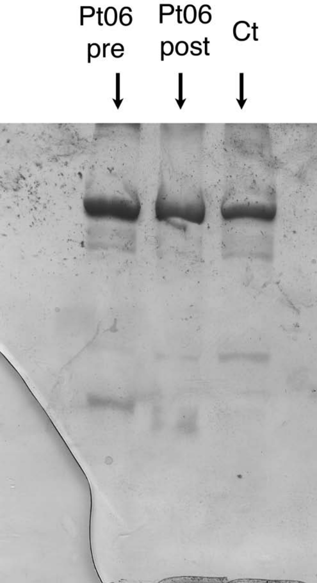

Supplement: Supplementary file 4 — Source Data for Figure 3 [file EMMM-7-1513-s002.pdf]
